# On-surface synthesis of planar π-extended [12]cycloparaphenylene

**DOI:** 10.1038/s42004-022-00720-5

**Published:** 2022-08-22

**Authors:** Victoria Richards

**Affiliations:** Communications Chemistry, https://www.nature.com/commschem

**Keywords:** Surface chemistry, Nanoscale materials

## Abstract

[*n*]cycloparaphenylenes feature extensive *para*-conjugation that leads to useful electronic and optoelectronic properties, but their strained topology prevents their conversion into planar macrocycles. Now, on-surface coupling of cleverly designed precursors affords planar *π*-extended [12]cycloparaphenylene.

The bottom-up, atomically precise synthesis of carbon nanostructures enables the tailoring of their electronic properties at a molecular level. [*n*]cycloparaphenylenes ([*n*]CPPs)—closed rings of phenylenes linked in the *para* position—possess desirable *π*-electron delocalization along the carbon backbone, but the high strain in these systems prevents their *π*-extension into either larger or planar structures. Now, a collaboration led by Sabine Maier and Andreas Görling at Friedrich-Alexander-Universität Erlangen-Nürnberg and Konstantin Amsharov at Martin-Luther-Universität Halle-Wittenberg in Germany describes the on-surface synthesis of planar *π*-extended [12]CPP, featuring an all-armchair edge topology, whereby the peripheral phenylene units are solely *para*-conjugated (10.1038/s41557-022-00968-3)^1^.

Planarizing CPPs requires the introduction of a strong in-plane bend in what would otherwise be a straight edge. The team achieved this by designing bowl-shaped dibrominated indacenopicene precursors that contain the appropriate curvature for the paraphenylene backbone. These precursors were covalently coupled on a Au(111) surface—where the surface acts as both a support and a catalyst—*via* an Ullmann-type dehalogenative coupling followed by cyclodehydrogenation. Trimers, tetramers ([12]CPP) and pentamers form *via* *cis* coupling, while dominant *trans* coupling leads to chain structures (Fig. 1).

**Fig. 1:**
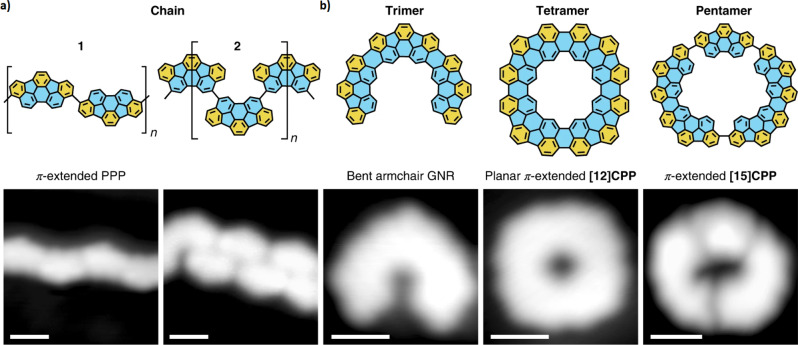
Atomically precise carbon nanostructures formed by Ullmann-type coupling of dibrominated indacenopicene precursors followed by cyclodehydrogenation on Au(111). *Trans* coupling leads to *π*-extended polyparaphenylene chains (**a**), while *cis* coupling affords bent armchair graphene nanoribbons (trimers), planar *π*-extended [12]CPPs (tetramers) and *π*-extended [15]CPP (pentamers) (**b**). Micrographs are high-resolution scanning tunneling microscopy images, with scale bars 1 nm. Reprinted by permission from Springer Nature: Nat. Chem., copyright 2022.

“The exclusive *para*-conjugation at the periphery of planar *π*-extended [12]CPP yields delocalized electronic states and facilitates a strong electronic communication along an extended *π*-system.”, explains Maier. Furthermore, planarization maximizes *p*-orbital overlap, contributing to a reduced electronic bandgap in comparison to conventional CPPs. Density functional theory calculations additionally find that [12]CPP features ring currents in its doubly charge configuration, affording global aromaticity.

Looking to the future, Maier hopes to experimentally characterize the unique electronic properties of such systems: “Synthesis strategies on insulting surfaces should be explored to decouple the molecular systems from the metal substrate electronically. Scanning probe measurements in the presence of a magnetic field could then facilitate the first direct visualization of ring currents at the atomic scale.” Furthermore, these properties could serve to make planar *π*-extended CPPs promising quantum materials.
